# Comparison of outcome and toxicity of postoperative intensity‐modulated radiation therapy with two‐dimensional radiotherapy in patients with soft tissue sarcoma of extremities and trunk

**DOI:** 10.1002/cam4.1919

**Published:** 2019-02-10

**Authors:** Jianyang Wang, Yongwen Song, Xinfan Liu, Jing Jin, Weihu Wang, Zihao Yu, Yueping Liu, Ning Li, Hui Fang, Hua Ren, Yuan Tang, Yu Tang, Bo Chen, Ningning Lu, Shunan Qi, Shulian Wang, Yexiong Li

**Affiliations:** ^1^ Department of Radiation Oncology, National Cancer Center/National Clinical Research Center for Cancer/Cancer Hospital Chinese Academy of Medical Sciences & Peking Union Medical College Beijing China

**Keywords:** conventional radiotherapy, extremity and trunk, intensity‐modulated radiation therapy, soft tissue sarcoma, survival

## Abstract

**Background:**

To compare the survival outcomes and late toxicities of postoperative intensity‐modulated radiation therapy (IMRT) with two‐dimensional radiotherapy (2D‐RT) for patients with soft tissue sarcoma (STS) of extremities and trunk.

**Methods:**

274 consecutive patients with nonmetastatic STS of extremities and trunk treated with postoperative IMRT (n = 187) and 2D‐RT (n = 87) were analyzed. Survival was calculated by using Kaplan‐Meier method. Independent prognostic factors were identified using Cox stepwise regression analysis for variables with a *P*‐value <0.1 in univariate analysis.

**Results:**

With a median follow‐up time of 58.1 months, 30 local recurrences, 66 distant metastases, and 40 deaths occurred. Compared to 2D‐RT group, the IMRT group had higher 5‐year local recurrence‐free survival (LRFS) (91.1% vs 80.8%; *P* = 0.029), distant metastasis‐free survival (DMFS) (80.0% vs 69.7%; *P* = 0.048), disease‐free survival (DFS) (75.2% vs 59.2%; *P* = 0.021), and overall survival (OS) (90.2% vs 81.0%; *P* = 0.029). Multivariate analysis showed IMRT was an independent favorable factor for LRFS, DMFS, DFS, and OS. For late toxicities, the patients in IMRT group enjoyed lower incidences of ≥Grade 2 joint stiffness (3.9% vs 12.3%; *P* = 0.041) and ≥Grade 3 fractures (0.0% vs 3.4%; *P* = 0.25) than those in 2D‐RT group. ≥Grade 2 Edema was similar between these two groups (4.8% vs 9.2%; *P* = 0.183).

**Conclusions:**

When compared with conventional techniques, postoperative IMRT seems to provide better LRFS, DMFS, DFS, and OS and less late toxicities in patients with STS of extremities and trunk, which should be offered as a preferred treatment.

## 1 INTRODUCTION

In the past 30 years, the management of soft tissue sarcoma (STS) had evolved from amputation and similar radical resection approaches to more conservative, function‐preserving treatment, in which radiotherapy (RT) played an important role. The evidence comes from two single‐institution, randomized trials^1^, ^2^ which demonstrated that radiotherapy in combination with limb‐sparing surgery has achieved better local control and similar overall survival as compared with surgery alone. However in the past, postoperative radiotherapy in extremity STS usually used two‐dimensional radiotherapy (2D‐RT) to large volumes, including the whole affected compartment of the limb with relatively large margins, inevitably getting surrounding normal tissue exposed,^3^ which is considered to increase the risk of severe late morbidity such as fibrosis, edema, joint stiffness, and fracture.^4^ Intensity‐modulated radiation therapy (IMRT) is a relatively new, but increasingly accepted, technology in radiation oncology that delivers radiation more precisely to the tumor. Recently, dosimetric studies showed that IMRT has the advantage of improving dose distribution to large tumor target while sparing normal tissue for STS (Table 1).^5^, ^6^

**Table 1:** Comparison of postoperative radiation techniques for soft tissue sarcoma of extremities and trunk

Institution/Authors	Technique (Number. of patients)	5‐y LC (%)	Edema (≥G2%)	Joint stiffness (≥G2%)	Fracture
Davis AM, et al^23^	2D‐RT/Conventional RT (56)	98.6	23.2	23.3	NS
MDACC^34^	2D‐RT (165)	88(10‐y)	NS	NS	
Holt GE, et al^29^	Conventional RT (172)	NS	NS	NS	9.8
MSKCC^11^	IMRT (63)	92	NS	NS	NS
MSKCC^12^	IMRT (41)	94	12.2	17.1	4.8
MSKCC^9^	Conventional RT (154) vs IMRT (165)	84.9 vs 92.4*	14.9 vs 7.9*	11.0 vs 14.5	9.1 vs 4.8 (≥G2)
Our hospital	2D‐ RT (87) vs IMRT (187)	80.8 vs 91.1*	9.2 vs 4.8	12.3 vs 3.9*	3.4 vs 1.1 (≥G2)*

2D‐RT, two‐dimensional radiotherapy; IMRT, intensity‐modulated radiation therapy; MDACC, MD Anderson Cancer Center; MSKCC, Memorial Sloan Kettering Cancer Center; NCIC, NCI Canada Clinical Trial Group Randomized Trial; NS, not specified.

* Statistically significant.

Although various studies have demonstrated the improved survival and decreased toxicity with IMRT in other malignant tumors,^7^, ^8^ only one study from Memorial Sloan Kettering Cancer Center (MSKCC)^9^ to date has assessed the differences between IMRT and conventional external‐beam radiation therapy in local recurrence (5‐year LR 7.6% vs 15.1%, *P* = 0.05) for patients with STS of the extremity. No other study had addressed the issue that whether such dosimetric improvements of IMRT can translate into reduction in complications and improved local control. In this large series of patients with STS of the extremity and trunk, we compared the survival outcome and toxicity of adjuvant IMRT and 2D‐RT after function‐preserving surgery in patients with primary localized STS of the extremities and trunk.

## 2 MATERIALS AND METHODS

### 2.1 Patients

Patients with STS treated with function‐preserving surgery and radiotherapy in our institution from January 2005 to December 2015 were identified. Two hundred and seventy‐four patients who met the following criteria were included in this study: tumor located in extremity or trunk, treated with postoperative IMRT (n = 187) or 2D‐RT (n = 87), and no previous radiotherapy. The exclusion criteria included those who underwent amputation, those with lymph node or distant metastasis at the time of presentation, and those whose radiation was performed outside our institution. Patients were staged according to the 7th AJCC staging system.^10^ For patients treated with recurrent tumor at presentation, stage was made according to recurrent disease instead of primary one.

### 2.2 Treatment

All patients received wide local excision. 250 patients (91.2%) had R0 resection (>1 mm margin), whereas 24 (8.8%) patients received R1 (≤1 mm margin or microscopic residual disease) or R2 (gross residual disease) resection.

Radiotherapy was administered 4‐6 weeks after surgery. For IMRT, the patients were immobilized and had computed tomography (CT) simulation. The clinical target volume (CTV) was defined as tumor bed plus 3‐4 cm margin in the superior and inferior directions, and 1.0‐1.5 cm margin in the medial and lateral directions, without expanding beyond the anatomy barrier. The surgical scar and drain sites were included in CTV. The first planning target volume (PTV1) was produced by expanding 0.5‐1.0 cm from CTV. The PTV2 was defined as PTV1 reduced by 3 cm in the superior and inferior directions. In first phase, a total dose of 50 Gy in 25 fractions was delivered to 95% of PTV1 with 6 MV X‐rays. In the second phase, 10‐16 Gy was boosted to 95% of PTV2. 16 patients received 10‐20 Gy boost with 6‐9 MeV electrons to the superficial boost target volume. All IMRT beams were arranged on one side of the extremity to spare a longitudinal strip of normal soft tissue.

For 2D‐RT, the patients were immobilized with customized device and simulated with fluoroscopy. No attempt was made to irradiate the entire muscle compartments or muscle bundles from origin to insertion. The entire circumference of an extremity was never treated, and care was taken to spare as much limb circumference, normal bone and joints as possible. Most patients were treated with high energy 6‐MV photons alone, usually with parallel opposed beams which were sometimes angled. In the first phase, RT field was designed to treat all areas at risk for tumor spread, encompassing the surgical bed/scar plus up to 5 cm margin in the cranio‐caudal direction and 2‐3 cm in the lateral direction, according to preoperative CT or MRI scans, and a dose of 50 Gy (100% to the isocenter) in 25 daily fractions over 5 weeks was delivered. In the second phase, a "shrinking field" technique was then used to treat 2 cm around the tumor bed and scar with 10‐16 Gy (100% to the isocenter) in five daily fractions during the sixth and seventh week of radiation therapy. Where considered more appropriate, 33 patients in the second phase were treated with an electron field.

Higher boost dose of 16‐20 Gy was delivered to the 24 patients with positive margins in the second phase.

### 2.3 End points and statistics

Overall survival (OS), disease‐free survival (DFS), local recurrence‐free survival (LRFS), and distant metastasis‐free survival (DMFS) were calculated from the date of the surgery. Local recurrence was defined as any recurrence in the primary site, irrespective of distant metastasis. Morbidity was defined as treatment‐related toxicities; those due to tumoral progression were excluded. It was assessed using the Common Terminology Criteria for Adverse Events (CTCAE) version 3.0.

The follow‐up schedule consisted of clinical evaluation including toxicity assessment every 3 months for the first 2 years and imaging of the primary and chest every 6 months, then every 6 months until 5 years, and then yearly.

Patients' demographic and clinico‐pathological characteristics were summarized through descriptive analysis. Continuous variables were described as means (SD) and compared using Student's *t* test. Qualitative variables were described as frequencies and percentages and compared using Fisher exact or chi‐square test. Survival time was calculated by using Kaplan‐Meier method. Differences in survival were tested by log‐rank test. Then, independent prognostic factors were identified using Cox stepwise regression analysis with 95% confidence interval (95%CI) for variables with a *P*‐value <0.1 in univariate analysis. Statistical analyses were performed using SPSS 22.0 software (SPSS Inc Chicago, IL). All P values are 2‐tailed, and confidence intervals (CIs) were calculated at the 95% level. A *P* value of <0.05 was considered statistically significant.

## 3 RESULTS

### 3.1 Clinical characteristics

Table 2 summarizes the demographic, tumor and treatment characteristics of both IMRT and 2D‐RT groups. The median age was 46 years (range, 5‐79), and the patient population was male dominant 163 (59.5%). There were more patients who were >50 years old (51.9% vs 32.2%, *P* = 0.003) and T2 tumors (55.1% vs 39.1%, *P* = 0.019) in IMRT group than that of 2D‐RT group. Patients with trunk STS were more likely to receive IMRT (81.1% vs 63.5%, *P* =* *0.005) than those with Extremities STS. The median radiation dose was 64 Gy (range, 50‐70) and 62 Gy (range, 50‐72) in 2D‐RT group and IMRT group, respectively. Other factors were comparable between the IMRT and 2D‐RT group in terms of median age, gender, histological types, tumor depth, grade, stage, margin status, and the use of adjuvant chemotherapy.

**Table 2:** Demographic and treatment characteristics of 187 patients with postoperative IMRT and 87 patients with postoperative 2D‐RT

Variable	IMRT	2D‐RT	*P*
	N (%)	N (%)	
Gender			
Male	116 (62.0)	47 (54.0)	0.235
Female	71 (38.0)	40 (46.0)	
Age			
Median years ± SD	50.9 ± 17.0	43.0 ± 15.8	0.039
>50 y	97 (51.9)	28 (32.2)	0.003
≤50 y	90 (48.1)	59 (68.8)	
Histology			
Malignant fibrous histiocytoma	43 (23.0)	17 (19.5)	0.633
Liposarcoma	43 (23.0)	14 (16.1)	
Synovial sarcoma	22 (11.8)	15 (17.2)	
Fibrosarcoma	33 (17.6)	20 (23.0)	
Rhabdomyosarcoma	3 (1.6)	1 (1.1)	
Others	43 (23.0)	20 (23.0)	
Location			
Extremities	127 (67.9)	73 (83.9)	0.005
Trunk wall	60 (32.1)	14 (16.1)	
Size			
Median diameters ± SD (cm)	6.0 ± 4.0	5.0 ± 4.0	0.111
≤5 cm	84 (44.9)	53 (60.9)	0.019
>5 cm	103 (55.1)	34 (39.1)	
Depth			
Superficial	53 (28.3)	25 (28.7)	1.000
Deep	134 (71.7)	62 (71.3)	
Stage (AJCC 7th)			
Stage I	47 (25.1)	23 (26.4)	0.900
Stage II	119 (63.6)	53 (60.9)	
Stage III	21 (11.2)	11 (12.6)	
Grade			
G1	47 (25.1)	23 (26.4)	0.880
G2	97 (50.3)	46 (52.9)	
G3	46 (24.6)	18 (20.7)	
Presentation			
Primary tumor	126 (67.4)	55 (63.2)	0.497
Recurrent tumor	61 (32.6)	32 (36.8)	
Resection			
R0 resection	171 (91.4)	79 (90.8)	0.823
R1/2 resection	16 (8.6)	8 (9.2)	
Adjuvant chemotherapy			
Yes	41 (21.9)	15 (17.2)	0.423
No	146 (78.1)	72 (82.8)	

2D‐RT, two‐dimensional radiotherapy; IMRT, intensity‐modulated radiation therapy.

### 3.2 Outcome and prognosis

**Figure 1:**
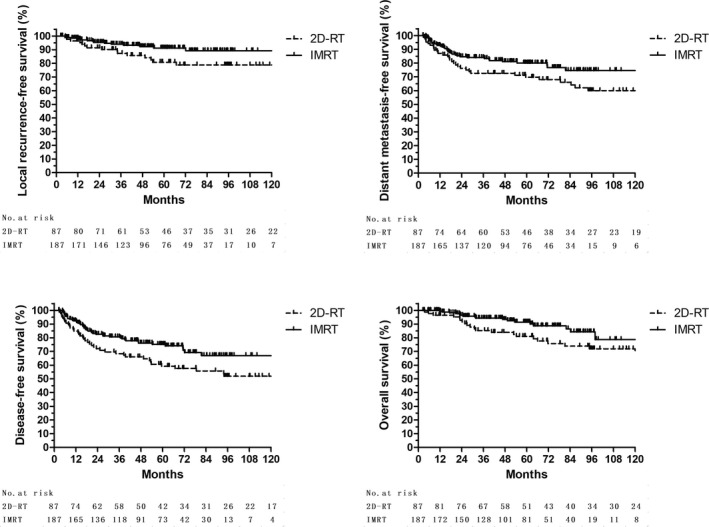
Kaplan‐Meier survival stratified by IMRT vs 2D‐RT

With a median follow‐up time for the cohort of 58.1 months (71.4 months for 2D‐RT group and 51.7 months for IMRT, *P* < 0.001), thirty patients had local recurrence (LR), sixty six patients developed distant metastasis (DM), and eleven patients had both LR and DM. Forty patients died and all from STS (Table 3). The 5‐year actuarial LRFS, DMFS, DFS, and OS rates for the cohort were 87.5%, 76.5%, 69.5%, and 87.0%, respectively. Compared to 2D‐RT group, the IMRT group had higher 5‐year LRFS (91.1% vs 80.8%, *P* = 0.029), DMFS (80.0% vs 69.7%, *P* = 0.048), DFS (75.2% vs 59.2%, *P *= 0.021), and OS (90.2% vs 81.0%, *P* = 0.029) (Table 3, Figure 1).

**Table 3:** Outcome of 187 patients with postoperative IMRT and 87 patients with postoperative 2D‐RT

Outcomes	IMRT	2D‐RT	*P*
	N (%)	N (%)	
Death	17 (9.1)	21 (24.1)	0.001
Events	46 (55.2)	39 (44.8)	0.001
Local recurrence	14 (7.5)	16 (18.4)	0.012
Distant metastasis	36 (19.3)	30 (34.6)	0.009
5‐y OS	90.2%	81.0%	0.029
5‐y LC	91.1%	80.8%	0.036
5‐y DMFS	80.0%	69.7%	0.048
5‐y DFS	75.2%	59.2%	0.021

DFS, disease‐free survival; DMFS, distant metastasis‐free survival; LC, local control; OS, overall survival.

Univariate analysis of prognostic factors for LRFS, DMFS, DFS, and OS was shown in Table 4. Larger tumor size was associated with poor LRFS and OS. For patients with tumor larger than 5 cm, the 5‐year LRFS and OS rate were 81.7% and 80.8%, respectively, compared with 92.9% (*P =* 0.015) and 92.5% (*P *= 0.014) for those with tumor 5 cm or less. Advanced disease stage also indicates poor OS. The OS were 92.7% for Stage I patients, 86.3% for Stage II, and 74.2% for Stage III (*P *= 0.039). Multivariate analysis revealed that 2D‐RT (HR = 2.30, [95% CI, 1.18‐4.48]; *P* = 0.014), large tumor (>5 cm) (HR = 2.26, [95% CI, 1.12‐4.55]; *P* = 0.023), and advanced disease stage (HR = 1.73, [95% CI, 1.01‐2.98]; *P* = 0.046) were independent risk factor of OS. Poor local control was found to be predicted by large tumor (>5 cm) (HR = 2.91, [95% CI, 1.31‐6.45]; *P* = 0.009) and 2D‐RT (HR = 2.58, [95% CI, 1.23‐5.38]; *P* = 0.012) (Table 4).

**Table 4:** Univariate and Multivariate COX Analysis for Prognostic Factor of Outcome in 187 Patients with Postoperative IMRT and 87 Patients with Postoperative 2D‐RT

Factor	Univariate analyses								Multivariate analyses							
	OS		LRFS		DMFS		DFS		OS		LRFS		DMFS		DFS	
	HR	*P*	HR	*P*	HR	*P*	HR	*P*	HR	*P*	HR	*P*	HR	*P*	HR	*P*
Female vs male	0.773	0.466	0.592	0.194	0.783	0.362	0.832	0.439	‐	‐	‐	‐	‐	‐	‐	‐
Age >50 y vs <50 y	1.566	0.178	1.254	0.564	2.237	0.002	2.172	0.001	‐	‐	‐	‐	2.225	0.002	2.144	0.001
Located in trunk wall vs extremities	1.686	0.145	1.222	0.646	0.772	0.408	1.083	0.761	‐	‐	‐	‐	‐	‐	‐	‐
T2 vs T1	2.600	0.014	2.987	0.007	2.743	<0.001	2.782	<0.001	2.257	0.023	2.907	0.009	2.692	<0.001	2.729	<0.001
Deep vs superficial	1.000	0.999	1.380	0.539	0.969	0.921	1.049	0.869	‐	‐	‐	‐	‐	‐	‐	‐
Stage III vs I + II	1.161	0.039	1.800	0.242	1.351	0.388	1.289	0.413	1.734	0.046	‐	‐	‐	‐	‐	‐
Grade 3 vs 1/2	1.104	0.804	1.230	0.635	1.222	0.487	1.086	0.752	‐	‐	‐	‐	‐	‐	‐	‐
Recurrent vs primary tumor	0.961	0.627	1.537	0.107	1.113	0.674	1.455	0.430	‐	‐	‐	‐	‐	‐	‐	‐
R1/2 vs R0 resection	0.844	0.782	2.127	0.171	1.942	0.265	0.801	0.608	‐	‐	‐	‐	‐	‐	‐	‐
ACT vs no ACT	0.820	0.656	0.481	0.230	1.361	0.284	0.526	0.392	‐	‐	‐	‐	‐	‐	‐	‐
2D‐RT vs IMRT	2.348	0.011	2.450	0.013	2.199	0.002	2.205	<0.001	2.301	0.014	2.583	0.012	2.227	0.002	2.232	<0.001

2D‐RT, 2‐dimensional radiotherapy; ACT, Adjuvant chemotherapy; R1 resection, resection with ≤1 mm margin or microscopic residual disease; R2 resection, resection with gross residual disease; T2, tumor>5 cm.

### 3.3 Morbidity

One patient in IMRT group and 2 in 2D‐RT group developed grade 4 acute dermatitis and wound complication after radiation. Late complications were shown in Table 5. No grade 5 toxicity occurred. The patients in IMRT group enjoyed lower incidences of ≥Grade 2 joint stiffness (3.9% vs 12.3%; *P* = 0.041) and ≥Grade 3 fractures (0.0% vs 3.4%; *P* = 0.25) than those in 2D‐RT group. ≥Grade 2 Edema was similar between these two groups (4.8% vs 9.2%; *P* = 0.183).

**Table 5:** Late morbidities of 187 patients with postoperative IMRT and 87 patients with postoperative 2D‐RT

Late morbidity	IMRT	2D‐RT	*P*
	N (%)	N (%)	
Fracture			
Grade 2	2 (1.1)	0 (0.0)	0.025
Grade 3‐4	0 (0)	3 (3.4)	
Edema			
Grade ≥2	9 (4.8)	8 (9.2)	0.183
Joint stiffness			
Grade ≥2	5 (3.9^a^)	9 (12.3^b^)	0.041

IMRT, intensity‐modulated radiation therapy; 2D‐RT, two‐dimensional radiotherapy.

a Analysis in 127 patients received IMRT with STS of the extremity.

b Analysis in 73 patients received 2D‐RT with STS of the extremity.

## 4 DISCUSSION

To our knowledge, our study is the first study to directly compare not only clinical survival outcomes, but also late toxicities of postoperative IMRT and 2D‐RT for STS patients, and our results demonstrated that IMRT provided better local control (5 year 91.1% vs 80.8%) and OS (5 year 90.2% vs 81.0%) and less severe late toxicities compared with 2D‐RT.

As a major advancement of high radiation techniques during the past decade, IMRT has been widely used in the clinical practice. A possible concern is that the distribution of dose is tight for IMRT compared with 2D‐RT, which might compromise subclinical or microscopic lesion coverage. However, preliminary clinical data from MSKCC showed an excellent local control rate with 5‐year LC 92%‐94% in a group of patients with locally advanced extremity STS,^11^, ^12^ which is consistent with 87%‐96% achieved by 2D‐RT techniques^2^, ^13^, ^14^ and this result. Recently, Folkert et al from MSKCC reported that IMRT was associated with significantly reduced local recurrence compared with 2D‐RT (5‐year LR 7.6% vs 15.1%, *P* = 0.05) for STS of the extremity.^9^ On multivariable analysis, IMRT remained an independent predictor of reduced local recurrence. Our study has further confirmed that IMRT provided lower local recurrence (5‐year LR 8.9% vs 19.2%, *P* = 0.029) than 2D‐RT and was an independent predictor for better local control. 2D‐RT may have reduced accuracy of treatment versus IMRT with image guidance and 3D imaging. The concern was raised on tumoral miss with 2D technique; thus, its radiation fields should be more generous to avoid undertreating disease. Also, increasing use of MRI for target delineation and decision making in recent years for IMRT group might be helpful to improve the outcome.^15^

In the past, there was concern about application and generalization of IMRT, as IMRT is of tight dose distribution, an advantage in reducing RT morbidity to surrounding normal structures, might compromise tumor coverage. However, Cleator et al studied relapse patterns of STS and demonstrated that the most of patients relapse sites (68%) located within the primary tumor bed.^16^ A randomized trial defined target volume as the tumor bed plus a 2 cm margin for postoperative STS patients by using brachytherapy and showed local control with high‐grade sarcoma were similar to those of entire compartment irradiated, which indicated that the entire compartment may be not necessarily included in the PTV1.^1^ In clinical results, Alektiar et al showed IMRT could contribute to an excellent local control in a group of high‐risk STS patients.^9^, ^12^ Under certain circumstances, the tumor coverage may even be improved using IMRT.

However, there has been still no comparison between IMRT and 2D‐RT with long‐term follow‐up, in terms of the potential effects on survival. Whether better local control could benefit survival have yet to be established. Previous case‐control studies show no differences in local control, and overall survival unless dose escalation was used for other malignancies.^17^ This study first showed that IMRT with relatively tight margins did not compromise the LC, but improved 5‐year DMFS (80.0% vs 69.7%, *P* = 0.048), DFS (75.2% vs 59.2%, *P* = 0.021), and OS (90.2% vs 81.0%, *P* = 0.029) than 2D‐RT, despite that there are more patient in IMRT group with negative prognostic factors for DM and OS,^3^, ^17^, ^18^, ^19^, ^20^, ^21^ such as older age, larger tumor. IMRT achieved better target coverage and allowed more precise delivery of high doses to the target volume than conventional 2D‐RT, as a result, better local control and overall survival.

Currently, the main motive to choose IMRT over non‐IMRT is its advantage to reduce toxicities. For many years, the conventional RT technique for limb STS was limited to two‐dimensional beams to entire compartment of the limb, covering the entire affected compartment with large margins and covering surrounding normal tissue,^3^, ^22^ thus resulted in high rates of morbidities in spite of its excellent LC. Even though, reducing the morbidities of postoperative RT should not rely on lowering the prescription dose, but minimizing the target volume and sparing the surrounding critical normal tissue. The risk of late toxicity following RT for extremity sarcoma has been shown to increase as the radiotherapy field size increases.^23^ IMRT offers the opportunity to reducing the radiation field, better conform to the target volume with smaller margin and delivers radiation more precisely to the target volume while sparing the surrounding critical normal tissue.^23^, ^24^, ^25^

Our previous study showed IMRT had lower RT‐related late morbidities as fibrosis, edema, and joint stiffness.^26^, ^27^ Further, in the current study, we found significantly higher incidence of late complications, with 9.2% ≥Grade 2 edema, 12.3% ≥Grade 2 joint stiffness and 3.4% ≥Grade 2 bone fracture in patients treated with 2D‐RT than those treated with IMRT. Similarly, Davis et al reported that 23.3% of patients had edema and 23.3% had joint stiffness when treated with 2D‐RT.^23^ Cannon et al showed that the 20‐year chronic radiation‐related limb complications rate was 13% in patients with primary lower extremity STS.^28^ The overall fracture rate ranged from 1.2% to 6.3%.^12^, ^28^, ^29^, ^30^ In recent years, three groups using primarily IMRT for sarcomas in extremities reported similar Grade 2 or greater toxicity rates. These toxicity rates are likely more representative of current practice: edema (5‐11%), joint stiffness (5.5‐14.5%).^31^, ^32^ With respect to fracture rates, Dickie et al defined RT dose constraint which, when achieved, keep fracture rates <2%,^33^ which is consistent with 1.1% ≥Grade 2 fracture rates in our study, while Grade 3‐4 fracture (3.3%) was only found in patients received 2D‐RT.

The limitations of this study relate to its retrospective nature with its inherent biases. Second, as the median follow‐up time was relatively shorter for patients in IMRT group compared with those in 2D‐RT group. Taking together, this may overestimate the survival and local control and underestimate the morbidity of radiation, especially for the patients in IMRT group. Even though, 51.7 months follow‐up time for current IMRT cohort is longer than those in previous study.^9^, ^11^, ^12^ Thus the conclusion of this study still stands. At last, the treatment in this study was postoperative, while STS is shifting toward preoperative radiotherapy with subsequent surgery based on the NCIC randomized trial results.^14^, ^23^

In conclusion, according to our data, compared with conventional techniques, postoperative IMRT provided better LC and OS and less severe late toxicities in patients with STS of extremities and trunk. Further evaluation in the prospectively randomized settings would be recommended to confirm the clinical advantages of IMRT over 2D‐RT.

## CONFLICT OF INTEREST

None declared.
